# Study on active capacity and detergent application potential of low-temperature alkaline serine protease produced by new strain *Exiguobacterium indicum* 1.2.3

**DOI:** 10.1186/s40643-023-00701-z

**Published:** 2023-11-08

**Authors:** Ahmet Kati, Gamze Balci

**Affiliations:** 1 Department of Biotechnology, Institute of Health Sciences, University of Health Sciences Turkey, 34668 Istanbul, Türkiye; 2 Experimental Medicine Research and Application Center, University of Health Sciences Turkey, 34668 Istanbul, Türkiye

**Keywords:** *Exiguobacterium indicum*, Alkaline protease, Low-temperature detergent, Industrial enzymes, Enzymatic activity

## Abstract

Microorganisms have long captivated researchers for their potential to produce enzymes with diverse industrial applications. Efficient production of proteases from new strains is crucial as these enzymes play a vital role in breaking down protein bonds, enabling their use in industrial applications. Therefore, a novel *Exiguobacterium indicum* 1.2.3 was isolated (Istanbul, Turkiye) and characterized in this study. This strain produced alkaline serine protease, which works in lower temperatures (20–40 °C) with casein as a specific substrate. The protease was utterly stable for 3 h at 30 °C. The enzyme was also highly stable in the pH range of 8–11. The optimum activity was obtained at pH 10. The crude enzyme activity was enhanced by various metal ions and retained 147%, 125%, 124%, and 117% of its activity within 1 mM Ca^2+^, Mn^2+^, Cu^2+^, and Mg^2+^, respectively. The crude enzyme was inactive with phenylmethylsulfonyl fluoride, indicating a serine residue on the active side. The enzyme exhibited a significant proteolytic effect in the presence of surfactants and oxidizing agents. The addition of Tween 80, Triton X-100, and sodium perborate improved enzymatic activity up to 135%, 109%, and 105%, respectively. According to the washing results, the crude enzyme effectively removed the blood on different types of standard pre-stained textiles at 30 °C. In conclusion, *Exiguobacterium indicum* 1.2.3 is a promising candidate for protease production, with its diverse applications spanning various industrial sectors, particularly detergents.

**Graphical Abstract:**

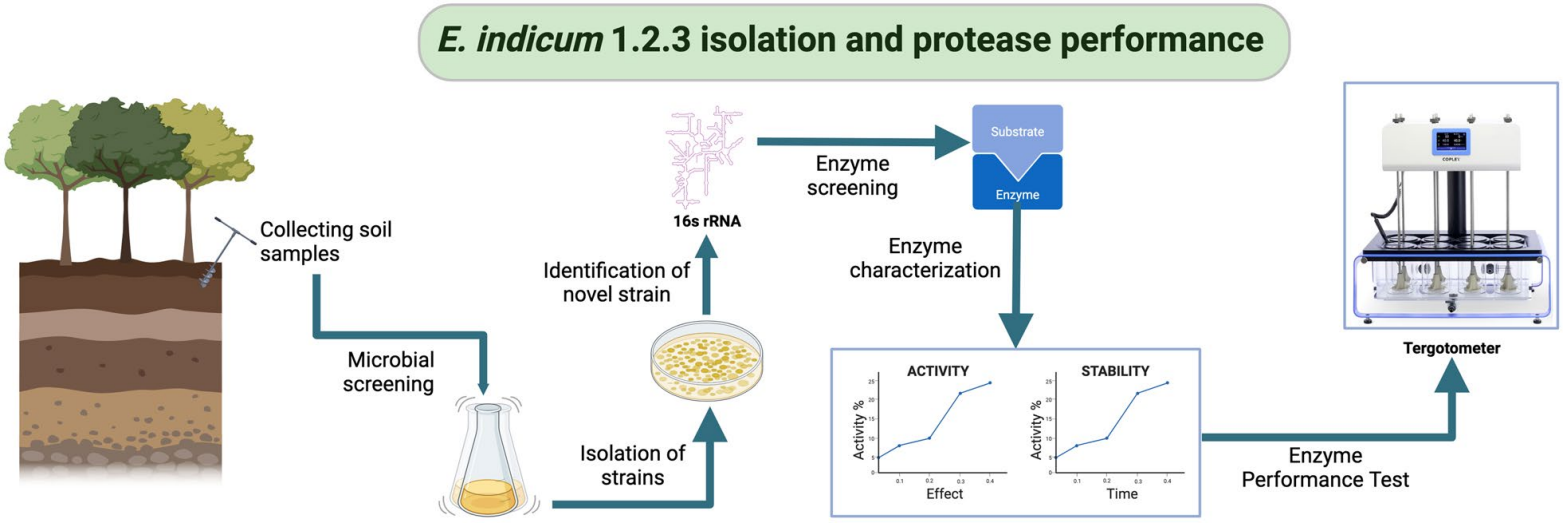

## Introduction

*Exiguobacterium* genus was first identified by Collins et al. (1983), as a Gram-positive, non-sporing, facultatively aerobic, alkaliphilic, and orange-colored bacteria (Chen et al. 2017). These group bacteria are isolated from soils, sediments, seawater, permafrost, glaciers, industrial waste, and hydrothermal vents. This bacterial family comprises 25 genera and can survive in various environmental conditions (D. Zhang et al. 2021). One of the unique features of this bacterium is its ability to produce an extremely stable protease that can work in a wide range of temperatures and pH levels. This protease has garnered attention in the biotech industry due to its potential to catalyze various reactions.

Industrial enzymes are obtained from microorganisms instead of animal or plant-based enzymes. Microbial-based enzymes have high activity, easy modification capacity, undesirable intermediate products, high stability, and cheap production (Raveendran et al. 2018). Therefore, the potential of microorganisms to rapidly produce enzymes enables this group of organisms to take the first place in meeting industrial demand. In addition, the possibility of identifying organisms that can produce biotechnologically more valuable and efficient enzymes encourages researchers to search for new enzyme-producing microbial strains (Ben Bacha et al. 2018). For example, within the extensive array of enzymes synthesized significantly by Bacillus species, proteases hold paramount importance due to their varied industrial uses; notably, subtilisin finds application as an additive in household detergents (Degering et al. 2010).

On the other hand, *Escherichia coli* is a bacterium well-known for its protease secretion and is commonly utilized in scientific research and biotechnological production. It facilitates the breakdown of hemoglobin through the heme-binding protein (Otto et al. 1998).

Bacterial proteases are widespread due to their extracellular nature and high production yield. However, their effectiveness is limited by various physicochemical factors such as enzyme instability at high temperatures and extreme pH, the presence of organic solvents, anionic surfactants, oxidizing agents, and the need for co-factors (Olajuyigbe and Falade 2014). Previous studies have determined that the bacterial isolate belongs to the *Exiguobacterium indicum* spp. produces the maximum amount of protease with a single carbon and nitrogen source (Hakim et al. 2018). Also, *Exiguobacterium* spp. has been confirmed to have a degradation and metabolizing effect. These results show strong evidence that members of the genus *Exiguobacterium* could utilize and metabolize various proteins and polysaccharides from environments (Zhang et al. 2021).

Detergent additives containing enzymes have been widely used since 1914 (Emon et al. 2020). Alkaline proteases are the main enzymes of the detergent, and their combination with different components enhances the washing effect. Alkaline protease is an endopeptidase highly active in a neutral-to-alkaline pH range. It is also known as a serine protease (Zhang et al. 2023).

Currently, there is a lot of focus on identifying alkaline proteases that can function effectively across a broad temperature range (Oberoi et al. 2001).

The washing effect of detergent can adapt to varying conditions with protease. A detergent enzyme must maintain stability even in the presence of oxidizing agents and bleaches. Unfortunately, many enzymes available in the market fail to meet this essential requirement. Exploring microbial diversity has immense potential for discovering novel enzymes with substantial commercial applications (Y. Zhang et al. 2023).

In the literature, there were minimal studies on *Exiguobacterium indicum*-based protease (Emon et al. 2020; Hakim et al. 2018; Kumar et al. 2018). However, it has exceptional protease properties. This study aimed to determine the extracellular protease production capacity, stability, and washing performance for the detergent industry of *Exiguobacterium indicum* 1.2.3 (*E. indicum* 1.2.3), which is a novel isolated strain from environmental soil samples, Istanbul.

## Materials and methods

### Bacterial strain and chemicals

*E. indicum* 1.2.3, a Gram-positive, spherical, orange pigmented with a smooth surface, was isolated from the soil samples in Kayisdagi, Istanbul (N 40° 58′ 26′′; E 29° 9′ 19′′) in 2013. The strain was identified using 16S rRNA gene sequencing (1472 bp) and submitted to NCBI with a Genbank accession number KF684939. This strain was first isolated from Turkiye.

Luria Bertani broth was obtained from Sigma-Aldrich. All the analytical grade chemicals, surfactants, and metals were purchased from Sigma, Himedia, ISOLAB Chemicals, Merck, and Neogen. Liquid laundry detergents were procured from the local manufacturer, Seba Kimya San. AS. The ELGA PureLab water system was used for preparing the solutions and buffer.

### Cultivation and media

*E. indicum* 1.2.3 was kept in a stock solution of 10% Nutrient broth and 10% glycerol. The culture was revived in a 10 mL Luria Bertani (LB) broth medium in a 50 mL Erlenmeyer flask and then streaked on LB agar. The alkaline protease production was achieved by using the following basal medium content (g/L): 0.5; glucose, 1.5; casein, 2; Na_2_SO_4_, 1; MgSO_4_.7H_2_O, 0.5; KH_2_PO_4_, 0.5; K_2_HPO_4_. The medium pH was adjusted to 7.5. This medium was added %1 (*v*/*v*) inoculant and incubated at 37 °C overnight at an orbital shaker with 150 rpm.

### Purification of crude protease

The cell-free supernatant was separated from the overnight culture through centrifugation at 14,000 rpm for 10 min at 4 °C using a refrigerated centrifuge (Allegra x-30r, Beckman Coulter, USA). This supernatant was precipitated with 50% ammonium sulfate at 4 °C (Maruthiah et al. 2013). The lysate was suspended within 0.1 M Tris–HCl buffer, pH 8.2. The precipitate was used for enzyme stability studies and application in detergent washing processes.

### Protease activity assay

The modified method of Hammami et al. (2017) was used to estimate protease activity. When protease breaks down casein, it releases various peptide fragments, including the amino acid tyrosine. The presence of free tyrosine triggers a reaction with Folin’s reagent, resulting in the formation of a measurable, blue-colored product. This product's absorbance is quantified at 660 nm using the BioTek Synergy Neo2 multi-mode spectrophotometer (Agilent, USA). To assess the extent of proteolytic activity, a calibration curve using a tyrosine standard is established to determine the quantity of liberated tyrosine (Beg and Gupta 2003; Senthilkumar et al. 2005).

Proteolytic activity was measured using 100 µL crude enzyme and 150 µL of 1% (*w*/*v*) casein as a substrate in 0.5 M Tris–HCl buffer (pH 8.2). The mixture of enzyme and substrate was incubated at 37 °C for 30 min. The reaction was terminated by adding 650 µL 1.2 M trichloroacetic acid (TCA). For negative control (blank), the mixture of substrate and TCA was used. Test and blank samples were put into the ice bath to eliminate the precipitation before being centrifuged at 8200 rpm for 10 min. After the centrifugation, 600 µL supernatant from each test and blank samples were blended with 0.5N Bradford reagent and mixed thoroughly. The absorbance was measured at 660 nm to find the one-unit protease activity according to the tyrosine standard graph. Various tyrosine standard solutions spanning 5 to 50 μg/mL concentrations were meticulously created using deionized water and the 0.18 mg/mL L-tyrosine stock solution. A single unit of proteolytic activity was established as the quantity of enzyme preparation needed to free one umol (181 µg) of tyrosine from casein in one minute, under pH 8.2 and 37 °C.

### Effect of pH on *E. indicum* 1.2.3 enzyme activity and stability

The influence of pH on protease activities was investigated across a pH spectrum of 3.0 to 12.0 at a temperature of 40 °C. The crude enzyme was incubated for 1 h at 40 °C to assess pH stability using diverse buffer solutions. The remaining enzymatic activities were evaluated under standard assay conditions. The following buffer systems were utilized with precision: 100 mM acetate buffer to cover pH 3.0 to 6.0; 100 mM sodium phosphate buffer for pH 7.0; 100 mM Tris–HCl buffer for pH 8.0; 100 mM glycine–NaOH buffer ranging from pH 9.0 to 11.0; and 100 mM KCl–NaOH buffer spanning pH 12 to 13.

### Effect of temperature on *E. indicum* 1.2.3 enzyme activity and stability

Temperature's impact was analyzed across the spectrum of 20 °C to 80 °C, measuring protease activity at pH 10. The enzyme preparation was exposed to varying temperatures, spanning 30 °C to 60 °C, for 120 min to monitor thermal stability. At specific time intervals, samples were extracted, and their residual activities were quantified using enzyme assay conditions—the unheated crude enzymes served as the control, indicating 100% activity.

### Effect of metal ions and inhibitors on *E. indicum* 1.2.3 enzyme activity

The impact of diverse metal ions (5 mM) on protease activities was explored through the introduction of divalent (Co2 + , Ca2 + , Cu2 + , Fe2 + , Mn2 + , Mg2 + , Ni2 + , Zn2 +), monovalent (Na + and K +) metal ions, and inhibitors (phenylmethylsulfonyl fluoride (PMSF), ethylene diamine tetra acetic acid (EDTA)) into the reaction mixture. Activity without metal ions served as the baseline at 100%.

### Effect of surfactants and oxidizing agents

The impacts of certain surfactants (SDS, Tween-80, TritonX-100) and oxidizing agents (hydrogen peroxide, sodium hypochlorite, sodium perborate) on enzyme stability were investigated by preliminary incubating the crude enzyme preparation at 30 °C for 1 h. The remaining protease activity was gauged at pH 10. The activities of the crude enzymes, subjected to analogous conditions without any additives, were considered 100%.

### Washing performance in detergent formulation

For washing performance, the homemade tergotometer was used. The tergotometer is a laboratory-scale multi-washing apparatus that assesses detergent components such as perfumes, fragrances, soaps, detergents, dyes, and surfactants. This equipment replicates the actions of an agitator-based washing machine, conducting washing processes within its six designated beakers (wash vessels) while maintaining controlled conditions of temperature and agitation speed. Each reservoir possesses a liquid capacity of 1000 mL. The temperature can be elevated to a maximum of 80 °C, and the rotational speed (rpm) is adjustable within the spectrum of 10 to 300 rpm. In the context of this investigation, the tergotometer's agitator speed was configured at 80 rpm, the temperature was adjusted to 30 °C, and the complete 1000 mL capacity was employed. The tergotometer experiments encompass the use of stained cloths and clean ballast cloths.

During the washing process, protease-specific stained fabrics were utilized: EMPA 111 (cotton stained with blood), EMPA 116 (cotton stained with blood/milk/ink), and EMPA 117 (Polyester/cotton, 65/35, stained with blood/milk/ink). The crude enzyme was added 0.5 and 1 g/L to the commercial ultra-white laundry liquid detergent without any enzyme. Also, only crude enzyme (1 g/L) was added to the washing condition to check its alone effect. The commercial ultra-white detergent was also used for the positive control, which contained the protease and other enzymes.

Each reservoir contained three stained fabrics and a 10 × 10 cm towel. The washing duration was set at 40 min. Post-washing, the stained fabrics were subjected to a 10-min rinse in 1 L tap water. Following rinsing, all stains were allowed to air dry at ambient temperature. The dried stains were ironed, and readings were taken using a Konica Minolta CM-700d spectrophotometer. Untreated stained fabrics were considered baseline references, and the reduction in stain intensity achieved after washing was quantified as a percentage using the provided equation:

$${\text{\% }}\,\,\,{\text{SR = (}}{{\Delta {\text{E}}} \mathord{\left/ {\vphantom {{\Delta {\text{E}}} {\Delta {\text{E}}_{0} }}} \right. \kern-0pt} {\Delta {\text{E}}_{0} }}) \times 100.$$

ΔE calculates the color distinction between the washed and unwashed cloth, whereas ΔE0 evaluates the variation between a standard clean cloth sample and the fabric before washing. The SR is referring to the stain removal percentage.

### Statistical analysis

The data for protease activity at various time intervals, pH levels, temperatures, and inhibition studies were analyzed using means, standard deviations, and ANOVA to assess their significance. The correlation between growth and enzyme activity at different time intervals was also determined. The p-value is less than 0.05. All experiments were conducted independently in triplicate.

## Result and discussion

### Microorganism and protease activity

**Fig. 1:**
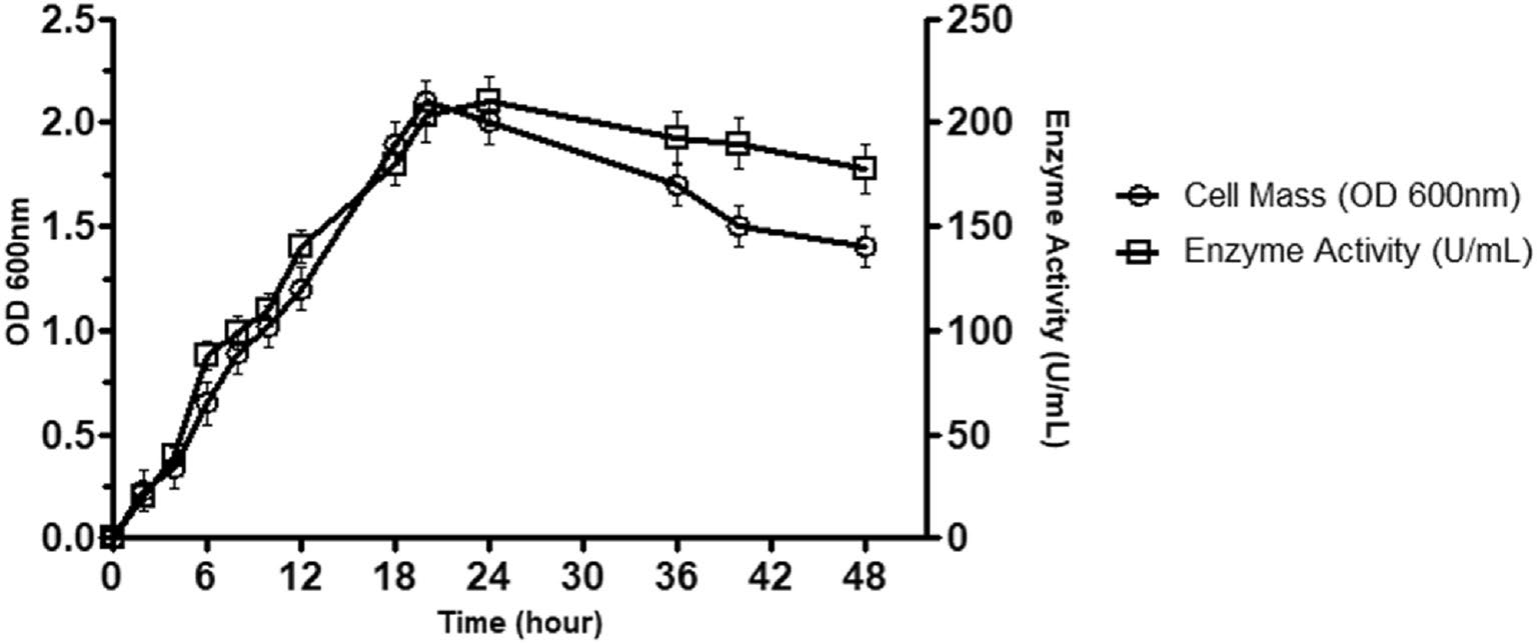
Growth curve and protease activity profile of *E. indicum* 1.2.3. All experiments were performed in triplicate with calculated mean standard deviation

*E. indicum* 1.2.3, producing highly stable alkaline protease, was newly isolated from soil samples and identified based on 16S rRNA gene sequencing. Protease activity was measured according to the tyrosine standard. The maximum growth rate and enzyme activity were measured in a basal medium. Protease activity and cell mass increased until 20 h of the log phase (Fig. 1). At the beginning of the stationary phase, the protease activity reached the maximum level. As the culture came to the late stationary phase, both protease activity and cell mass started to decrease because of the limitation of nutrients, increasing toxic waste of cells, and production of protease in the culture medium. Similar results were observed in the study of Hakim et al. (2018), who discovered the same profile for *E. indicum-*based protease activity.

### Characterization of *E. indicum* 1.2.3 protease

Discovering the proteolytic capacity of this bacterial enzyme was important for industrial applications under different environmental conditions.

### Effect of pH on enzyme activity and stability

**Fig. 2:**
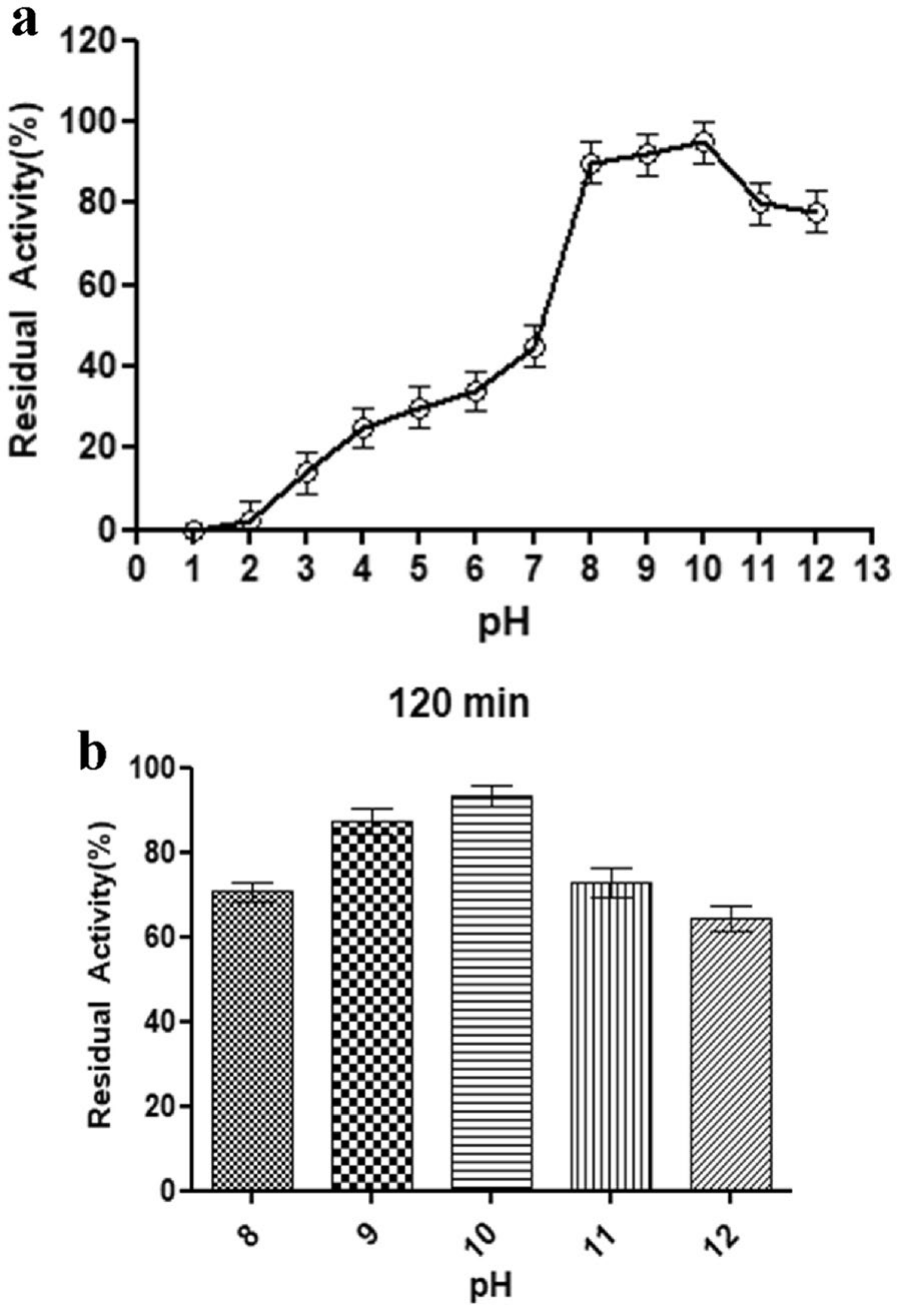
Effect of pH on protease activity (**a)** and stability (**b)**

*E. indicum* 1.2.3 has shown protease activity between pH 3–12. The optimum activity was observed in alkaline conditions at pH 10 (Fig. 2a). The relative pH stability was obtained at pH 10, which had more than 90% for 120 min, also at pH 9, 11, and 12, more than 70% was retained activity (Fig. 2b). This result, consistent with the previous studies, suggests that *E. indicum* produced the most significant quantity of protease under alkaline pH conditions. *E. indicum* AKAL 11 strain showed maximum protease activity at optimum pH 9. Another strain of *E. indicum* TBG-PICH-001 produced the maximum protease at pH 10 (Hakim et al. 2018; Kumar et al. 2018).

The high pH range stability increases the potential applications for different industries such as leather, textile, and wastewater treatment (Li et al. 2013; Sani et al. 2017; Sun et al. 2019).

### Effect of temperature on enzyme activity and stability

**Fig. 3:**
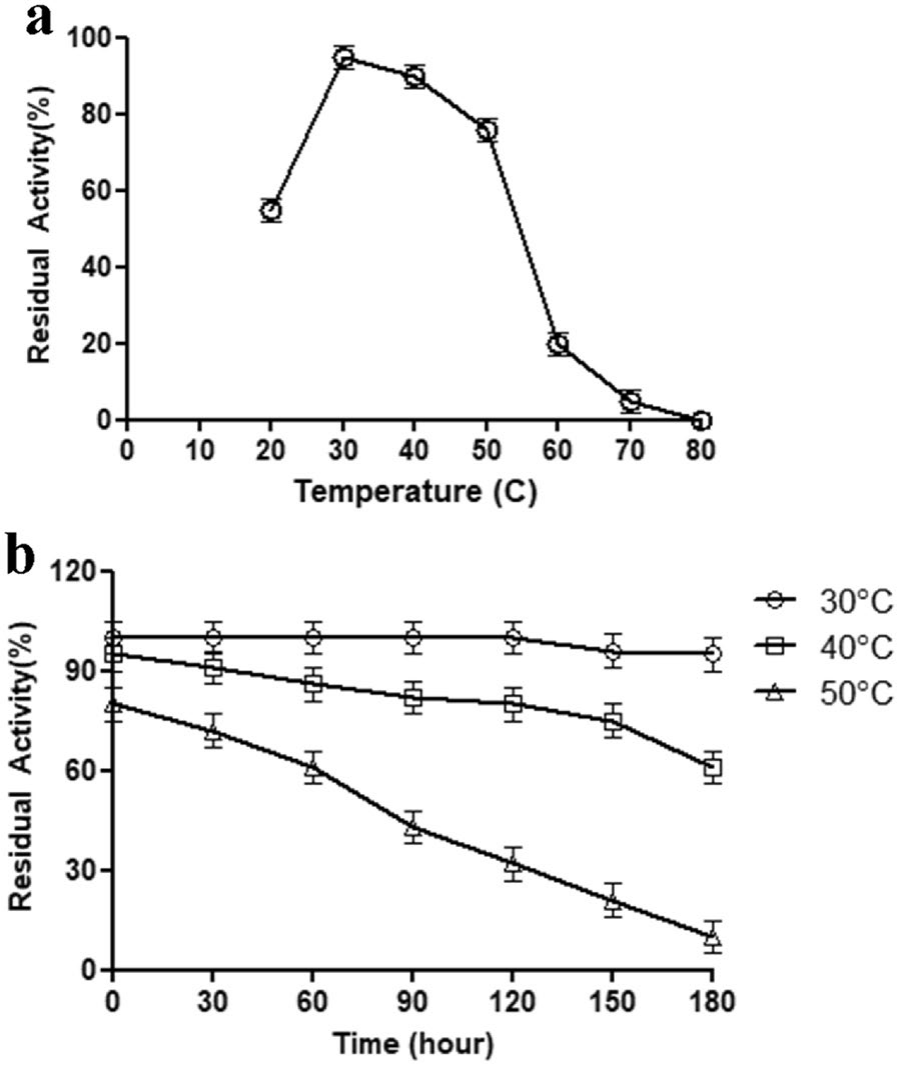
Effect of temperature on protease activity (**a)** and stability (**b)**

The alkaline characteristic protease showed maximum activity at 30 °C at pH 10 in 100 mM glycine–NaOH buffer solution. The protease showed thermal activation between 20 and 30 °C. The residual protease activity was kept around 75–85% between 40 and 50 °C. Above 60 °C, the protease undergoes thermal inactivation (Fig. 3a).

The thermal stability was performed at 30, 40, and 50 °C for 180 min according to residual protease activity. At 30 °C, the maximum protease activity was kept for 3 h. It had a positive effect on application conditions such as laundry washing environment. For 40 °C, the enzyme activity was evaluated at more than 80% for 2 h. Finally, for 50 °C, the proteolytic capacity was protected for one hour at around 60% and then decreased to less than 50% after 90 min (Fig. 3b).

Suwannaphan et al. (2017) showed that alkaline serine protease has a similar thermal profile range for activity. Almost identical results were obtained with *E. indicum* AKAL 11 and *E. profundum* BK-P23 for the optimum temperature was reported at 30 °C (Anbu et al. 2013; Hakim et al. 2018).

*E. indicum* 1.2.3 produces psychotropic protease, essential for the low-temperature detergent industry. Washing at lower temperatures allows fabric care, and the clothes look good for longer. Also, the low temperatures save energy with the help of the no-heating water, which has a critical environmental impact (Laitala and Jensen 2010; Vojcic et al. 2015).

### Effect of metal ions on enzyme activity

**Fig. 4:**
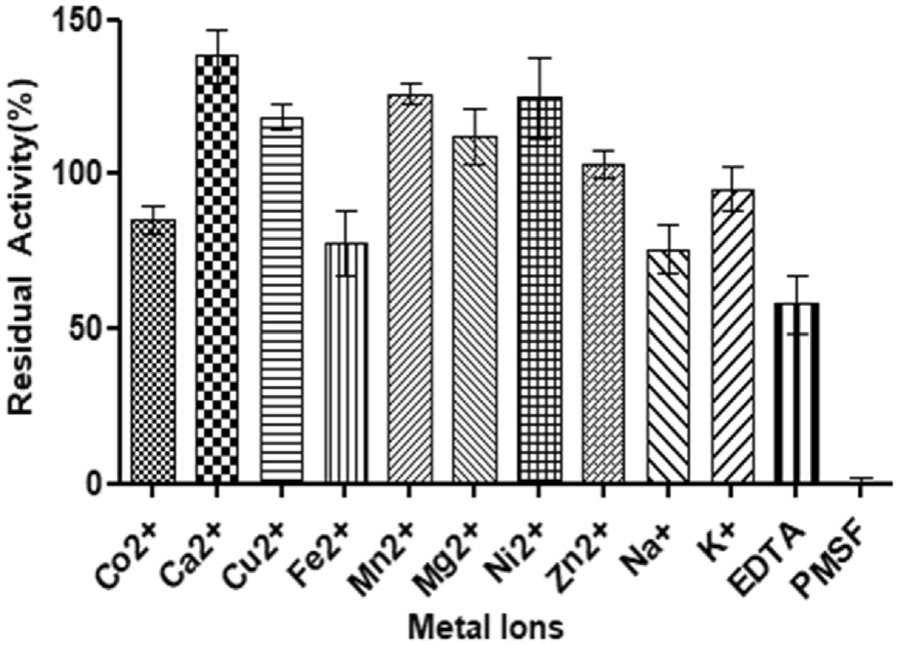
Effect of metal ions and inhibitors on protease activity

Protease activities were effectively assessed for their reaction to different metal ions. The results in Fig. 4 demonstrate that the addition of divalent Ca^2+^, Mn^2+^, Ni^2+^, Cu^2+^, and Mg^2+^ at 1 mM significantly boosted the proteolytic activity of *E. indicum* 1.2.3 by 47%, 25%, 24%, 17%, and 15%, respectively, when compared to the activity without metallic ions. The findings indicate the crucial role of ions in preserving the enzyme's active conformation. These results are similar to literature such as Hammami et al. (2018) found that Ca^2+^, Mn^2+^, and Mg^2+^ increased protease activity by 56%, 38%, and 23%, respectively.

Conversely, *E. indicum* 1.2.3’s enzyme activity was negatively affected in the presence of Fe^+2^, Co^+2^, and Na^+^ by 24%, 18%, and 11%, respectively. Suwannaphan et al. (2017) reported that adding Fe^2+^ was causing a decrease in serine protease relative activity by nearly 45%.

For inhibitory screening, PMSF completely inhibits enzyme activity at 1 mM, indicating that the enzyme is a serine protease with a serine residue in its active site. EDTA partially inhibits protease activity, suggesting a metal ion is required for substrate hydrolysis.

### Effect of surfactants and oxidizing agents on enzyme activity

**Fig. 5:**
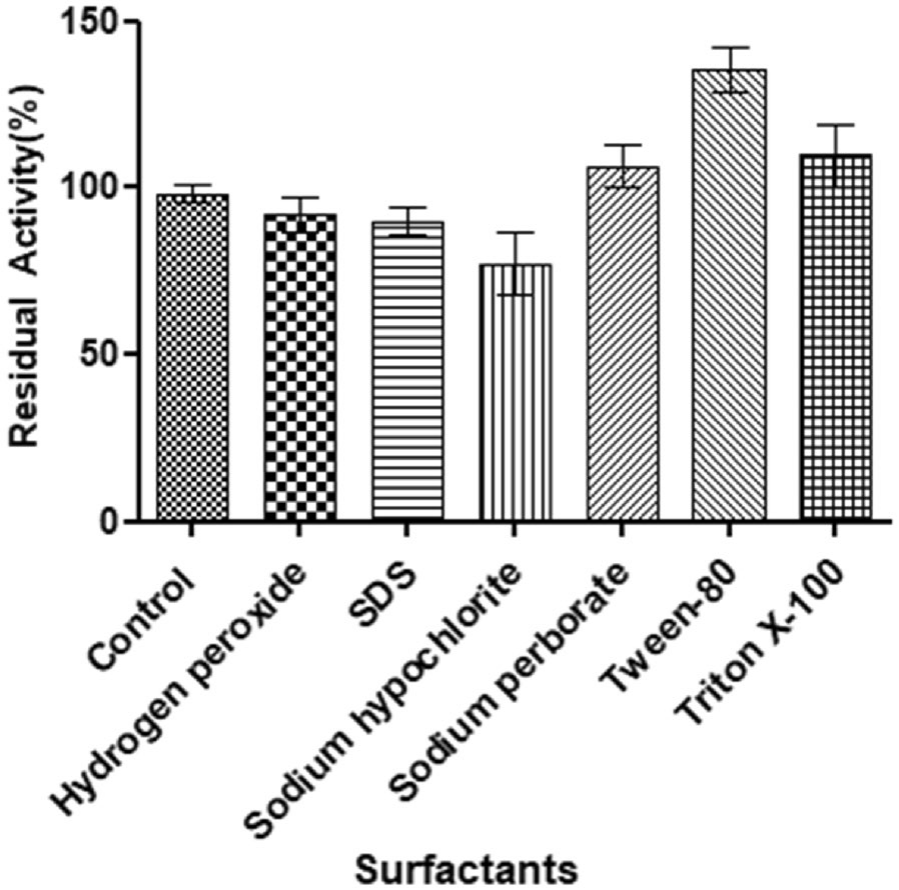
Effect of surfactants and oxidizing agents on protease activity

*E. indicum* 1.2.3 protease was assayed in the presence of oxidizing agents and reported in Fig. 5. After an hour of incubation, the crude enzyme preparation impressively maintained over 94% of its residual proteolytic activities even in the presence of 1% anionic surfactant (SDS). Ghorbel et al. (2014) findings were confirmed as *Streptomyces flavogriseus* HS1A increased residual protease activity to 18.6% in the presence of 0.5% SDS. Also, Gupta et al. showed that protease from *Bacillus mojavensis* was also stable in 1% SDS.

Under the effect of the non-ionic surfactants, crude protease activity markedly increased at 1% presence of Tween 80 and Triton X-100, 140%, and 110% of their initial activity, respectively. Suwannaphan et al. (2017) presented that the protease activity was unaffected in the presence of 0.5 and 1% Tween 20, Tween 80, and Triton X-100 and showed more than 80% activity.

The stability of protease was tested in the presence of oxidants. The protease showed highly stable activity with 1% sodium hypochlorite (> 75%), hydrogen peroxide (> 90%), and sodium perborate (> 105%). The feature of this protease can provide an opportunity for using an oxidant-stable enzyme in detergent formulations. In a study, a protease from *Pseudomonas aeruginosa* showed good compatibility with oxidizing agents (Grbavčić et al. 2011). Hammami et al. (2017) showed that sodium perborate did not affect protease activity.

Enzymes that are stable towards oxidizing agents and SDS are rare in wild-type microorganisms.

Enzyme-based detergents now use rDNA and protein engineering to create bioengineered enzymes that are more stable and efficient. Mutations have made protease preparations better suited for varying wash conditions and resist high temperatures and oxidizing agents (Beg and Gupta 2003).

### Washing performance in detergent formulation

**Fig. 6:**
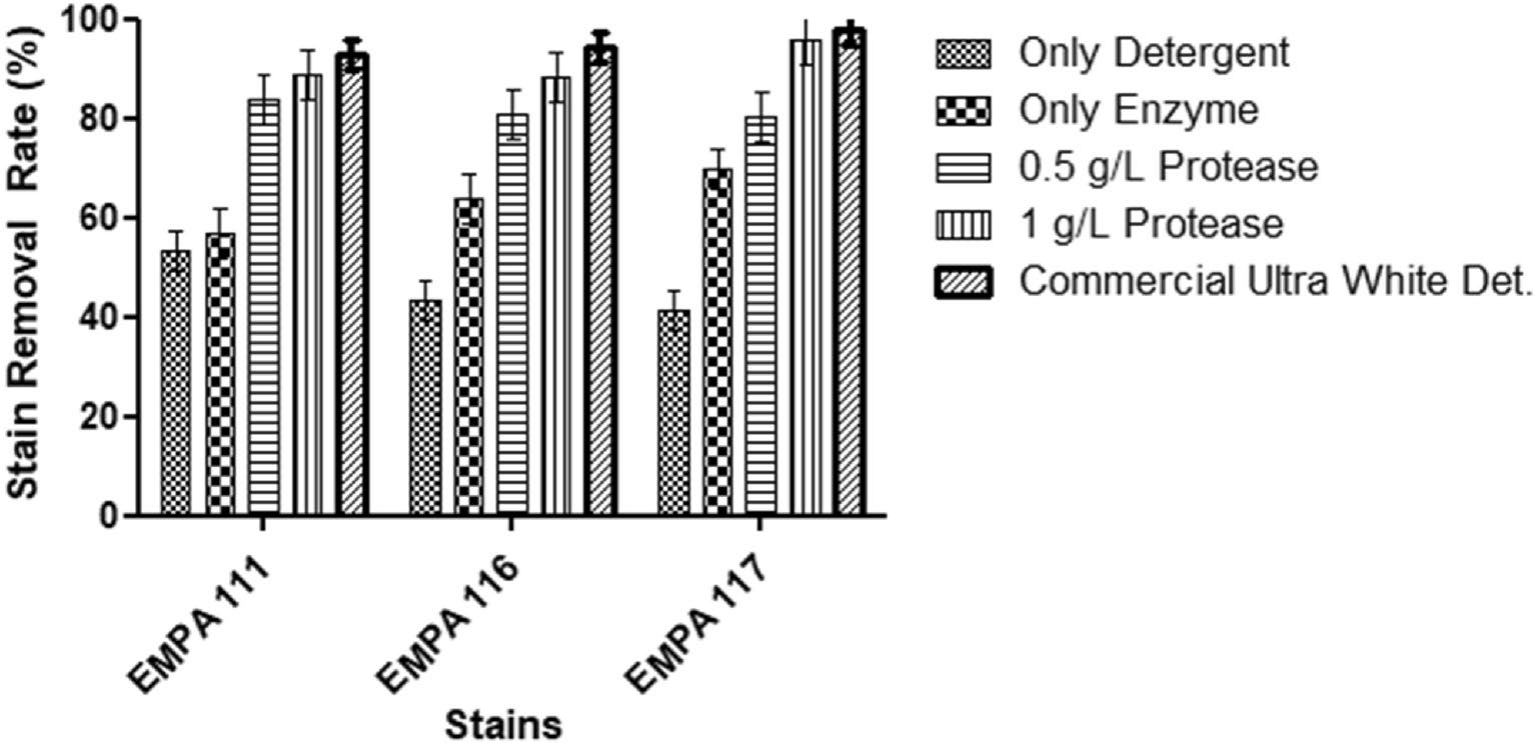
Enzyme washing performance in detergent formulation

Because of the urbanization progress, the detergent industries are continually growing, and formulations need more specific and powerful enzymes. The efficacy of the enzyme as a detergent additive was put to the test by washing protease-sensitive standard stains, which were EMPA 111 (cotton soiled with blood), EMPA 116 (cotton soiled with blood/milk/ink), EMPA 117 (polyester/cotton, 65/35, soiled with blood/milk/ink) at low temperatures (30 °C). After the washing test, the results, the removal rate of stain, was measured as a textile-specific spectrophotometer. The results are shown in Fig. 6. According to the results, the protease was very effective in detergent formulation against the stains as compared to the use of either only detergent or only enzyme. The *E. indicum* 1.2.3 protease showed promising activity for further studies when comparing the commercial ultra-white detergent washing performance.

According to the wash results, only detergent performance is limited because of the lack of enzymes. The 0.5 and 1 g/L enzyme-containing formulations showed > 80% and > 85% stain removal performance, respectively. In a previous study, an alkaline protease from *E. indicum* AKAL 11 showed good compatibility with different commercial detergent solutions (Emon et al. 2020). However, in this study, there was not any washing performance study.

## Conclusion

Industrial applications are evolutionary changing to replace eco-friendly solutions and enzymes instead of chemical catalysts. Therefore, finding new microorganisms that can produce novel and versatile enzymes for different applications is essential.

This is the first study to explore the novel *E. indicum* 1.2.3’s protease and its potential applications. According to the results, this protease is an alkaline serine, low temperature activated, highly stable under challenging conditions, and promising washing performance. The detergent formulation is a prime arena benefiting from the enzymatic potential of *E. indicum*-derived proteases. The enzymes offer enhanced stain removal and fabric care, promoting more eco-friendly and efficient detergent products. The thermostability and pH tolerance exhibited by *E. indicum* 1.2.3 proteases bolster their applicability in diverse washing conditions. Exploring its enzymatic potential broadens our understanding of microbial capabilities and contributes to the sustainable advancement of enzyme-based technologies, fostering greener and more effective solutions for modern challenges.

## Data Availability

All data generated or analyzed during this study are included in this published article.

## Abbreviations

*E. indicum*: *Exiguobacterium indicum*
PMSF: Phenylmethylsulfonyl fluoride
rRNA: Ribosomal ribonucleic acid
NCBI: National center for biotechnology information
LB: Luria Bertani
NaOH: Sodium hydroxide
HCl: Hydrochloric acid
TCA: Trichloroacetic acid
KCl: Potassium chloride
EDTA: Ethylene diamine tetra acetic acid
SDS: Sodium dodecyl sulfate
ANOVA: Analysis of variance
